# Deciphering the Diversity in Bacterial Transporters That Salvage Queuosine Precursors

**DOI:** 10.3390/epigenomes8020016

**Published:** 2024-04-25

**Authors:** Samia Quaiyum, Yifeng Yuan, Paul J. Kuipers, Maria Martinelli, Marshall Jaroch, Valérie de Crécy-Lagard

**Affiliations:** 1 Department of Microbiology and Cell Science, University of Florida, Gainesville, FL 32611, USA; megh1710@gmail.com (S.Q.); yuanyifeng@ufl.edu (Y.Y.); pkuipers@ufl.edu (P.J.K.);; 2 eSTEAMed Learning Inc., Maitland, FL 32751, USA; 3 Department of Oral Biology, College of Dentistry, University of Florida, Gainesville, FL 32610, USA; 4 Genetic Institute, University of Florida, Gainesville, FL 32611, USA

**Keywords:** queuosine, queuine, transporter, tRNA, salvage, biogeography, phylogenomic

## Abstract

Queuosine (Q) is a modification of the wobble base of tRNA harboring GUN anticodons with roles in decoding accuracy and efficiency. Its synthesis is complex with multiple enzymatic steps, and several pathway intermediates can be salvaged. The only two transporter families known to salvage Q precursors are QPTR/COG1738 and QrtT/QueT. Analyses of the distribution of known Q synthesis and salvage genes in human gut and oral microbiota genomes have suggested that more transporter families remain to be found and that Q precursor exchanges must occur within the structured microenvironments of the mammalian host. Using physical clustering and fusion-based association with Q salvage genes, candidate genes for missing transporters were identified and five were tested experimentally by complementation assays in *Escherichia coli*. Three genes encoding transporters from three different Pfam families, a ureide permease (PF07168) from *Acidobacteriota* bacterium, a hemolysin III family protein (PF03006) from *Bifidobacterium breve*, and a Major Facilitator Superfamily protein (PF07690) from *Bartonella henselae*, were found to allow the transport of both preQ_0_ and preQ_1_ in this heterologous system. This work suggests that many transporter families can evolve to transport Q precursors, reinforcing the concept of transporter plasticity.

## 1. Introduction

While the multifaceted functions of tRNA modifications in translation have been recognized for over four decades, only recently has it become apparent that these modifications may serve pivotal regulatory roles across various model systems from bacteria to humans [1]. In addition, technical advancements in the analytical techniques for detecting and quantifying many tRNA modifications have allowed systems-wide investigations into their functional significance, positioning this area of study within the epitranscriptomic domain [2,3,4,5].

Queuosine (Q) is a modification found at the wobble base (position 34) of tRNAs that decode NAC/U codons in most bacteria and eukaryotes. Although its role in decoding accuracy and efficiency has been well established, the effects of Q vary with the specific codon and organism, and these differences are not fully understood [6]. The discovery of most Q metabolism genes combined with the development of different analytical tools, such as 3-(Acrylamido) phenylboronic acid (APB)-based affinity-based gels, LC-MS/MS analysis of bulk tRNAs, and next-generation-based sequencing methods (reviewed in [6]), have led to the recent realization that this modification could also play regulatory roles in virulence and oxidative stress in bacteria by affecting the translation efficiency of specific genes enriched for specific codons [6].

**Figure 1:**
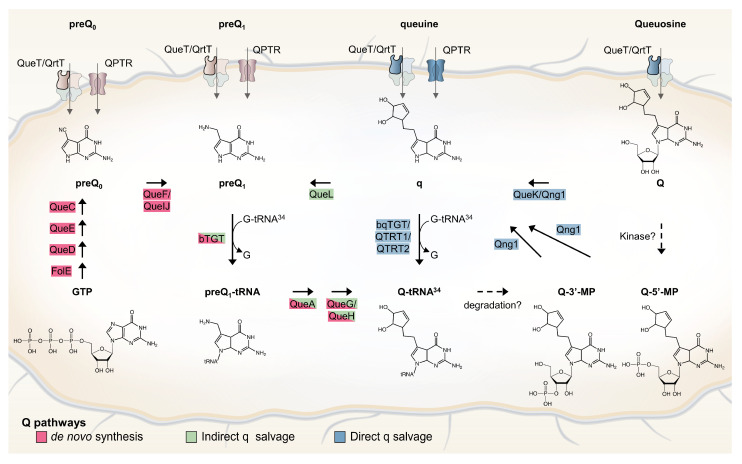
Bacterial Q de novo and salvage pathways. Q de novo synthesis pathway is depicted with enzymes that are shown in red. Indirect and direct q salvage pathways are depicted with enzymes that are shown in green and blue, respectively. Possible degradation and phosphorylation pathways are shown with dashed arrows. Among the four components of ECF transporters, only the substrate-specific transmembrane (S) component (QueT/QrtT) is labeled (bold border).

Only bacteria can synthesize Q de novo in a complex pathway that was recently extensively reviewed (Figure 1 and [6]). In short, GTP is the precursor molecule and, therein, its first step is shared with the tetrahydrofolate (THF) synthesis pathway. Four additional enzymes (QueD, QueE, QueC, and QueF) are required to make the 7-aminomethyl-7-deazaguanine (preQ_1_) precursor base that is exchanged with the guanine at position 34 of target tRNAs by tRNA-guanine (34) transglycosylase (bacterial TGT, bTGT). Additional tailoring enzymes (QueA, and QueG or QueH) finish the synthesis of Q on the tRNA molecule. Both preQ_1_ and its direct precursor 7-cyano-7-deazaguanine (preQ_0_) can be salvaged from the environment, with some bacteria relying only on the salvage route due to a lack of preQ_0_/preQ_1_ synthesis enzymes. The queuine (q) base can also be salvaged directly in some pathogenic bacteria, as well as in all eukaryotes. In these organisms, the bTGT enzyme has changed substrate specificity from preQ_1_ to q. Sources of the q bases are products of tRNA hydrolysis (Q, Q-3’MP, and Q-5’MP), and specific nucleoside hydrolases are required to liberate the q base from these precursors. Two, QueK and Qng1, have been experimentally characterized thus far. These enzymes are members of unrelated protein families and harbor different substrate specificities (Figure 1 and [7,8]). We recently discovered an indirect q salvage pathway in which a queuine lyase (QueL) enzyme regenerates a preQ_1_ intermediate that can then be used by canonical bTGT [7].

Like other purine derivatives, Q precursors (preQ_0_, preQ_1_, q, and Q) require specific transporters to be imported into the cell. Only two such transporter families have been characterized to date: the QPTR family, formerly named YhhQ, and the QueT/QrtT subgroups of the Energy-coupling Factor (ECF)-type transporters (see review in [6]). Substrate specificity differences have been observed between members of both transporter families. For example, QPTR from *E. coli* transports preQ_1_ and preQ_0_ but not q [9], while QPTR from *Chlamydia trachomatis* transports q but not preQ_1_ or preQ_0_ [7]. Similarly, one QrtT from *Clostridioides difficile* only transports preQ_1_, while the other transports preQ_1_, q, and Q [7]. However, the genes encoding Q precursor transporters have yet to be identified in most organisms, including all eukaryotes.

Recent studies have reinforced the importance of Q as a micronutrient [10,11], particularly for optimal brain function [12,13] and mitochondrial stress responses [14]. However, how the human host competes with organisms of the microbiome for Q precursors is poorly understood. Different members of the microbiome can generate Q de novo or act as preQ_1_/preQ_0_/Q scavengers [7,15]; hence, it is possible that competition between sympatric organisms could be observed for Q as it has been for B vitamins [16,17]. Moreover, Q supplementation does lead to an increased level of α-diversity in the intestinal microbiota [18]. The role of Q in microbiome composition, as well as the amount of Q produced and utilized by the gut microbiota, might affect the health of the host, as suggested by recent studies [19,20]. However, to construct an accurate model of Q exchange in the microbiome, all Q metabolism genes—including all missing transporters—must be identified, particularly in phyla most prevalent in the human gut and other specific microbial niches. Finally—as recent metagenomic and single-cell sequencing analyses have revealed regarding the temporal and spatial heterogeneity of bacterial communities inhabiting humans [21,22]—understanding the holistic biogeography of Q metabolism is critical to discerning how it may shape microbial communities and, therein, the emergent health of their hosts.

**Figure 2:**
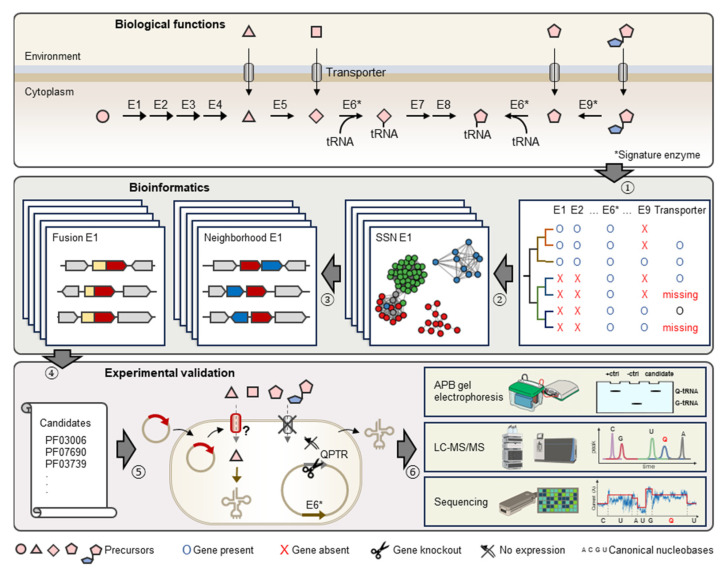
Schematic representation of comparative genomics-driven approach to identify novel Q transporters. (1) Identification of knowledge gaps in metabolic pathways, e.g., missing Q precursor transporters. (2) Construction of Sequence Similarity Networks (or SSNs) for signature enzymes in Q pathways, e.g., E6 (TGT) and E9 (Qng1/QueK). (3) Investigation of genomic neighbors and fusions for Pfam families of query signature enzyme. (4) Generation of candidates. (5) Expression of candidates in engineered *E. coli* strains lacking indigenous Q precursor transporter. (6) Detection, quantification, and localization of Q-tRNA using various technologies. Enzymes in Q biosynthesis pathway are represented by E1 to E9. Among them, E6 (TGT) and E9 (hydrolases) are signature enzymes of q salvage.

This study focuses on the reconstruction of Q metabolism for species in the gut and oral microbiome to evaluate the importance of Q precursor exchanges in these ecological niches and estimate the prevalence of unidentified Q precursor transporters. We then use the pipeline described in Figure 2 that combines comparative genomics and experimental techniques to identify and characterize missing transporter genes applied to the Q salvage pathway.

## 2. Results and Discussion

### 2.1. Q Makers and Users Are Spatially Distributed in the Human Gut and Oral Microbiomes

Previous reconstructions of Q pathways of the human microbiota used only de novo pathway genes and were restricted to a limited number of reference organisms [7,15]. To predict the importance of Q exchanges for different niches of the human microbiome, we analyzed the presence–absence patterns of all known Q synthesis and salvage genes in 13,027 genomes of the gut microbiome (Dataset S1) and 8547 genomes of the oral microbiome (Dataset S2).

**Figure 3:**
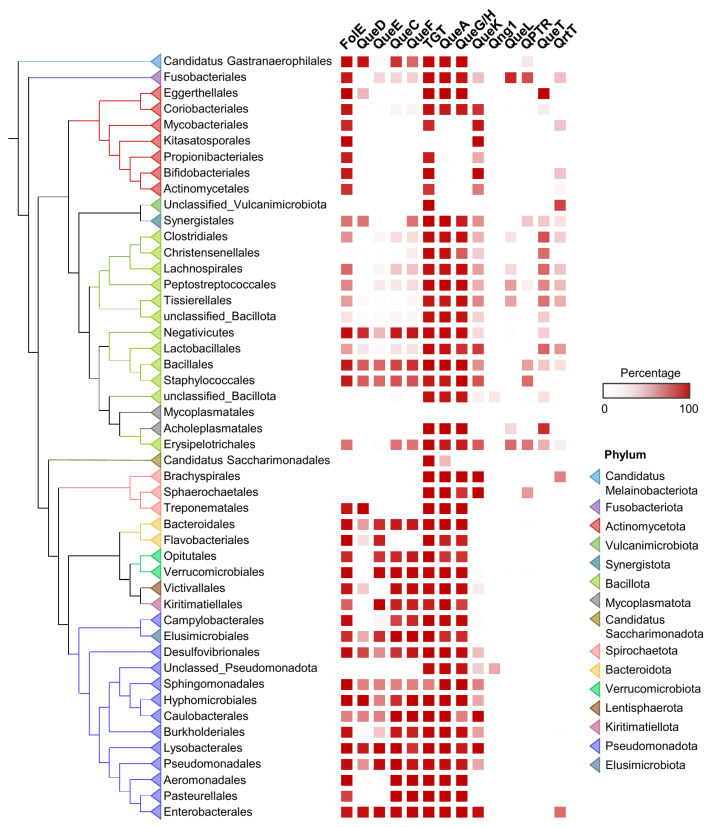
Distribution of Q pathway proteins in human gut microbiome. ML tree of concatenated ribosomal proteins of human gut microbiome genomes. Presence of Q pathway proteins in each taxon unit is indicated in red. Orders with less than 10 genomes are hidden or merged for better visualization.

In terms of Q synthesis, species of the gut microbiome fall within two major classes: one is enriched in Q makers, mainly Pseudomonadota (synonym Proteobacteria), that encode a full biosynthetic pathway (bottom half of the tree in Figure 3), and another enriched in Q precursor-users, composed mainly of Actinomycetota and Bacillota, lacking QueDECF proteins but still encoding bTGT, the signature enzyme in the pathway (top half of the tree in Figure 3). The types of salvage can be further specified within either class. For example, most Actinomycetota are predicted to salvage q as they lack QueA and QueH/QueG, whereas most Synergistales species are predicted to salvage preQ_1_/preQ_0_ bases. Additionally, it is clear that not all transporter genes have been identified, as many organisms predicted to salvage Q precursors lack homologs of QPTR or QueT/QrtT, a pattern seen quite strikingly in Propionibacteriales.

**Figure 4:**
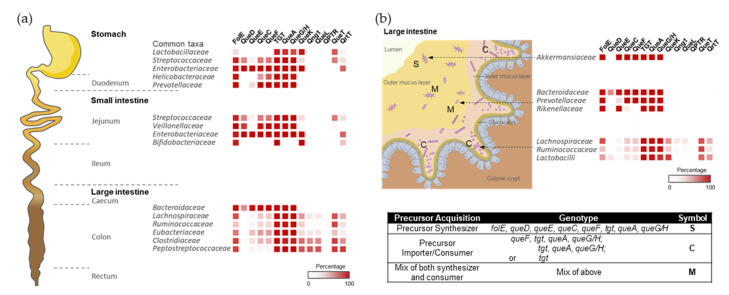
The colonization and spatial organization of Q pathways in the gut microbiota. (**a**) The distribution of bacterial composition and Q pathway proteins along the longitudinal axis of the human gut. (**b**) Bacterial compositions vary along the transverse axis of the mouse gut. The spatial distribution of gut microbiome species was adapted from references [22,28,29,30] with permission.

With this quite granular dataset in hand, we could set out to map Q metabolism on spatial species distribution maps (Figure 4). For example, Q users would be expected to be more abundant in the lower region of the gastrointestinal tract, such as the small intestine, where food is digested and may release Q precursors. Most species in the large intestine lack *queDECF* genes, instead harboring q salvage genes (QueKL) and transporters (Figure 4a), supporting our hypothesis.

Within each region of the gut, the host tissues, mucus layers, and luminal spaces present distinct habitats, leading to phylogenetic heterogeneity along the transverse axis of the gastrointestinal tract. We speculated that Q precursors could influence microbial organization at the micro-scale, like gradients of oxygen, AMPs, immune factors, and mucus density [22]. Indeed, in the mouse colon, members of the *Ruminococcaceae*, *Lachnospiraceae*, and *Lactobacilli* families are generally found as cecal crypt residents [23]; these species generally lack Q synthesis genes (Figure 4b). On the other hand, the loose outer mucus layer is colonized by commensals, including mucolytic bacteria, such as *Akkermansiaceae* [24] and some *Bacteroides* species [25], which can typically synthesize Q (Figure 4b). Therefore, a gradient of Q precursors may be present that decreases from the outer to the inner mucus layers and likely plays a role in shaping the composition of respective microbial communities.

**Figure 5:**
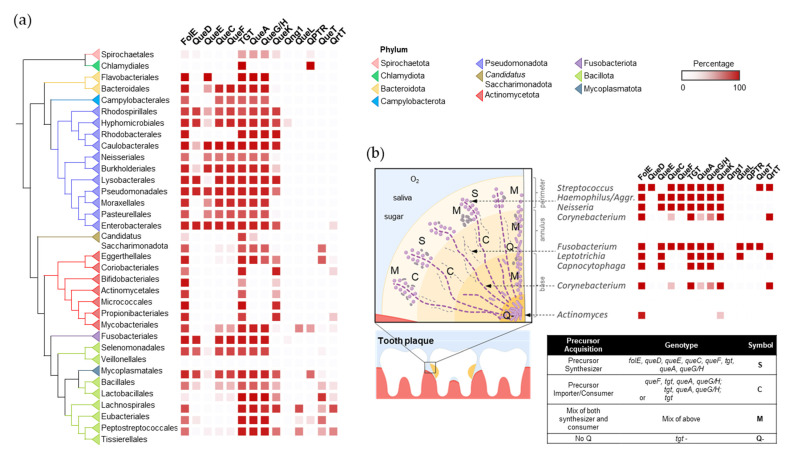
The distribution and spatial colonization of Q pathway proteins in the human oral microbiota. (**a**) Distribution of the Q pathway proteins in the human oral microbiome. (**b**) Spatial organization of the Q pathways in the human oral tooth microbiota. Aggr. is short for *Aggregatibacter*. The spatial distribution of oral bacteria was adapted from [21] with permission.

In the oral microbiome, the establishment of dental plaques is a sequential process starting with early colonizing species, such as *Streptococcus*, that cling to the tooth surface using specialized adhesins. Among early colonizing species, there appears to be no clear preference of Q salvage or Q synthesis, with examples like *Streptococcus gordonii* and *Streptococcus sanguinis* being salvagers, *Streptococcus mitis* encoding the full Q biosynthetic pathway, and *Actinomyces* not utilizing Q at all [26] (Figure 5 and Dataset S2). As dental plaque develops, defined structures, microenvironments, and specially organized collections of oral bacteria and yeast species begin to take shape, largely driven by the formation of extracellular matrices and biofilm species diversity, providing surfaces for other organisms to colonize [21]. The presence of oxygen correlates with Q utilization preferences, where oxygen-replete environments in the interior of the plaque show enrichments of Q precursor consumers (e.g., *Leptotrichia*, *Capnocytophaga*, and *Fusobacterium*) and organisms that do not utilize Q, including *Candida* species. Communities that colonize the exterior of the plaque are more likely to be composed of Q synthesizers and genera with mixed Q acquisition strategies. Organic acids produced by acidogenic organisms accumulate to form low-pH microenvironments within plaque layers. These acids can either encourage the growth of other acidophilic organisms or foster organisms that metabolize organic acids [27]. *Streptococcus mutans* is a key acid-producer and is also a Q synthesizer. After the founding of an acidic environment, species that can tolerate low pH, like the *Lactobacilli*, can colonize the plaque and primarily rely on the importation of Q precursors (Figure 5). Alternatively, the acid-metabolizing *Veillonella* species can help neutralize pH and are predominantly Q synthesizers. The dental plaque offers unique insights into synergism and competition between microorganisms, and, here, it is illustrated that Q precursors may be another resource that these species exchange and compete for.

### 2.2. Identification of Q Precursor Transporter Candidates through Comparative Genomics

Fusion and physical clustering analyses are powerful tools to identify missing genes in bacteria [31]. We hence used several of these strategies to identify missing Q precursor transporters. We first looked for protein fusions of transporter domains with bTGT (see Section 4). Three different transporter domains were identified as fused with bTGT (Table S1). One was the known preQ_1_/preQ_0_ transporter, QPTR (PF02592/COG1738/IPR003744). The other two were found to be transporters unique to different *Tsukamurella* species. Of these, Tpau_0550 (UniProt: D5USC3) belonged to the Major Facilitator Superfamily (MFS-1, PF07690), and the second was found to be a member of the MMPL family (PF03176), Tpau_4044 (UniProt: D5UNC0).

To complement the first, a second approach was implemented independently, in parallel, for the identification of putative Q precursor transporters. Specifically, this analysis examined the physical clustering of Q salvage signature genes, namely bTGT, Qng1, and QueK (see the pipeline described in Figure S1). Here, we used the Gene Neighborhood Network (GNN) tool in the Enzyme Function Initiative (EFI) suite [32] to survey neighbors of the targeted signature genes among bacteria. With this, 51 diverse transporter families were identified as potential candidates (Table S2). Because it was expected that organisms requiring Q salvage would likely necessitate the transport of Q precursors, these candidates were sorted using scores determined by each protein’s Q pathway profile as derived through comparative genomic analyses. Candidates with high scores (z-score ≥ 1) included known Q precursor transporters QPTR (PF02592) and QrtT (PF12822), as well as tentatively novel Q-relevant transporter families. Specifically, these new high-scoring candidates were as follows: the MMPL family (PF03176), Haemolysin-III (PF03006), the Fusaric acid resistance protein-like superfamily (PF13515), the sugar transporter-Major Facilitator Superfamily (PF00083-PF07690), and another ABC transporter family member (PF00005) (Table S2). A majority of these candidates were members of large transporter superfamilies with highly diverse subgroups that, historically, have been notoriously difficult to annotate.

**Figure 6:**
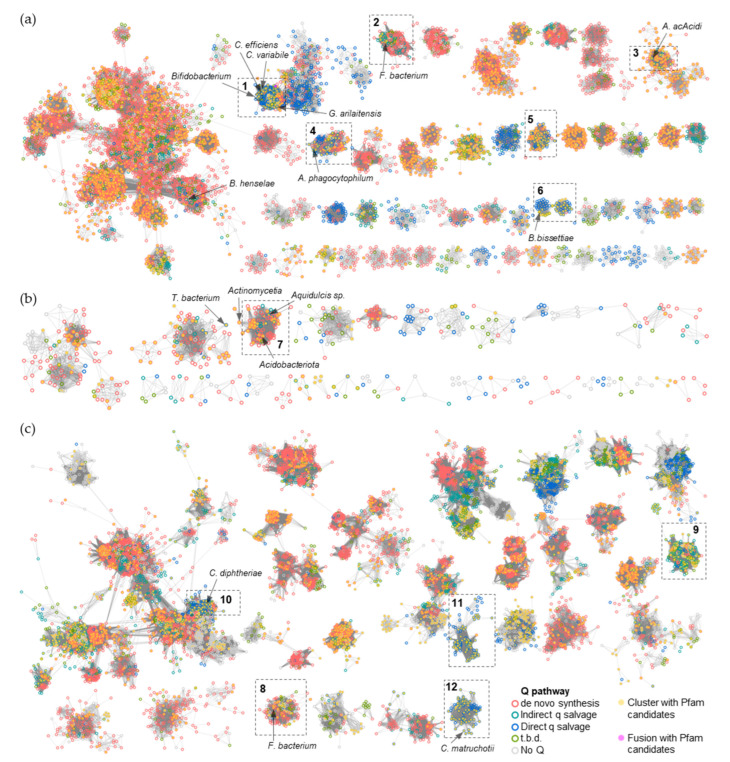
SSNs of bTGT, Qng1, and QueK proteins. (**a**) Each node in the network represents one or multiple bTGT proteins that share > 90% identity. An edge (represented as a line) is drawn between two nodes with a BLAST E-value cutoff of better than 10^–135^ (alignment score threshold of 135). (**b**) Each node in the network represents one Qng1 protein. An edge is drawn between two nodes with an alignment score > 90. (**c**) Each node in the network represents one or multiple QueK proteins that share no less than 90% identity. An edge is drawn between two nodes with an alignment score > 90. The nodes are circled based on the presence/absence of the other Q synthesis genes in the corresponding species. Species that do not encode bTGT are circled in gray. For better visualization, the solitary nodes and small clusters are hidden. Nodes are in full color when candidate transporters are present in the gene neighborhood (distance ≤ 3) or fused. Boxed clusters are analyzed further in Figure 7.

**Figure 7:**
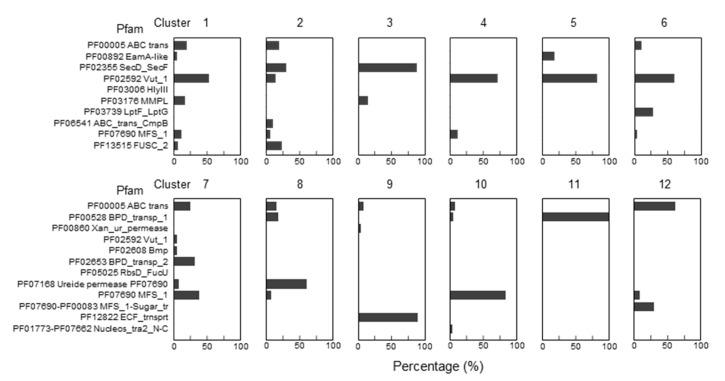
Enrichments of specific Pfam transporter families clustering with Q salvage genes. For each boxed cluster in Figure 6 (numbered 1 to 12), all transporter candidates present in each cluster (yellow nodes in the boxed cluster in Figure 6) were extracted and the percentage of candidate members of specific Pfam families was plotted for each cluster.

To narrow the list of candidates and to visually explore their physical clustering with Q salvage genes, we constructed protein Sequence Similarity Networks (SSNs) using EFI-EST (EFI Enzyme Similarity Tool) [32] for the bTGT, Qng1, and QueK protein families, coloring the sequence nodes based on the predicted Q pathway profile of the encoding organism and the presence of the 51 transporter candidates identified (Figure 6). Physical clustering between signature genes and transporter candidates occurred across each SSN generated (yellow nodes), confirming that organisms able to synthesize Q de novo may also encode transporters to salvage precursors, as previously observed in *E. coli* [9] (red-circled yellow nodes). Among the clusters in which transporters are present, we focused on those for organisms that are expected to require a q salvage pathway (blue- and dark green-circled nodes, 12 clusters boxed in Figure 6) and calculated the percentage of occurrence for each transporter candidate within that cluster. QPTR (PF02592) and QrtT (PF12822) dominated five out of the seven clusters in which they were present (Figure 7). MFS superfamily members (PF07690) were observed in two-thirds of the examined clusters, especially dominating clusters 7 and 10. Ureide permease (UPS, PF07168), which overshadowed all other candidate families in cluster 8, was prioritized for the same reason. Many organisms expected to require q salvage for Q synthesis were not found to encode a transporter that clusters with bTGT, Qng1, or QueK (blue- and dark green-circled open nodes), suggesting the possibility that other candidates have yet to be identified. Based on the physical clustering and predicted metabolic requirement of Q salvage, this analysis generated a final list of 10 candidates (Table 1) (Figure S2 and Table S3).

### 2.3. Members of Three Transporter Superfamilies Can Evolve to Transport preQ_0_ and preQ_1_

**Figure 8:**
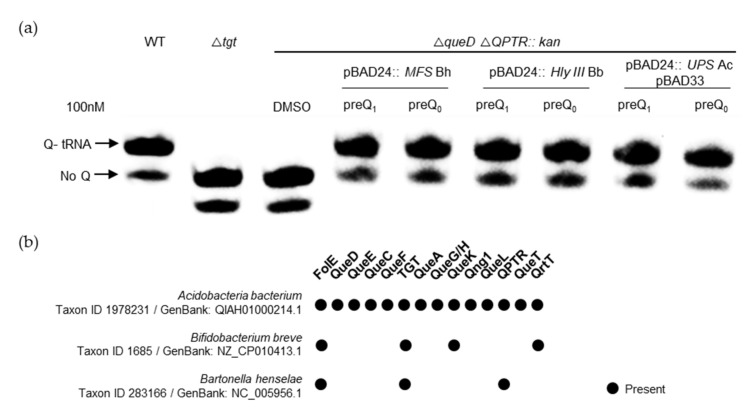
Experimental validation of preQ_0_/preQ_1_ transport activity. (**a**) tRNAs were extracted from WT, *tgt*, or *E. coli queD*-*yhhQ*/-QPTR double deletion mutants expressing the candidate MFS, Hly III, and UPS transporter genes, respectively, from *Bartonella heneselae* (Bh), *Bifidobacterium breve* (Bb), and *Acidobacteriota* bacterium (Ac), listed in Table 1, grown in minimal media in the presence of exogenous preQ_1_ or preQ_0_. The detection of Q-tRNA^Asp^_GUC_ was performed using the APB detection assay described in the Materials and Methods section, where Q-modified tRNAs migrate slower than unmodified tRNAs. (**b**) The presence of Q pathway genes in the organisms encoding the transporters tested in (**a**); data extracted from Dataset S3.

Having identified ten different candidate families, we selected five of them to target for experimental validation (Table 1). This was accomplished by expressing candidate transporter genes in strains of *E. coli* that are auxotrophic for preQ_0_/preQ_1_ due to the deletion of both the *queD* gene and *QPTR*, the latter of which results in an inability to transport either precursor. We found that the expression of members of three out of the five families tested was able to complement both the preQ_0_ and preQ_1_ transporter deficiencies of an *E. coli QPTR queD* double-deletion strain (Figure 8a and Figure S3). We cannot rule out that the lack of complementation with the genes encoding MMPL and LptG/F transporters was due to expression issues in a heterologous system. In the follow-up of these findings, we found that preQ_1_ could be transported even when present at low concentrations (5 nM) (Figure S4), suggesting that the expressed clones were encoded with preQ_1_ transporters of a particularly high affinity. However, it should be noted that the actual K_m_ values of each still require proper estimation.

Interestingly, Q pathway reconstructions were unable to predict that the MFS proteins of *Bartonella henselae* and *Bifidobacterium breve* would transport preQ_1_/preQ_0_, as they lack *queA* or *queG*/*H* genes (Figure 8b and Dataset S3). We have previously shown that the *B. henselae* QPTR and bTGT proteins preferred preQ_1_ as a substrate [33], and that preQ_1_ is found in tRNA suggesting that preQ_1_ and not Q was the final deazapurine modification present in tRNAs in this organism [33]. The importance of the preQ_1_ modification is reinforced by the discovery that a second preQ_1_ transporter of the MFS family is present adjacent to *tgt* in this organism (Figure S2). In the case of *B. breve*, our results suggest that preQ_1_ is transported and inserted into tRNA by a canonical bTGT enzyme. However, further experiments are needed to check if the Q pathway terminates here for this organism or whether another family of enzymes has yet to be discovered that may catalyze this remaining step. Finally, the Acidobacteriota UPS transporter candidate gene found encoded next to *qng1* was observed to transport preQ_0_/preQ_1_. This was not expected as Qng1 is only known to cleave the q base of Q or that of Q-MP precursors. Using a genetic setup previously implemented to validate q and Q transporters of different pathogenic bacteria [7], it seems that this UPS transporter does not transport Q; instead, it only transports q at very high, physiologically improbable concentrations (Figure S5).

### 2.4. Tentative Identification of preQ_1_ Transporter Subgroups in Three Transporter Superfamilies

The three new experimentally validated preQ_1_/preQ_0_ transporter genes presented by this work are members of notably large superfamilies, making it very difficult to confidently propagate any such annotations. Therefore, we opted to construct SSNs in an attempt to identify the potential isofunctional members of the preQ_1_-specific transporter subgroup of these families.

**Figure 9:**
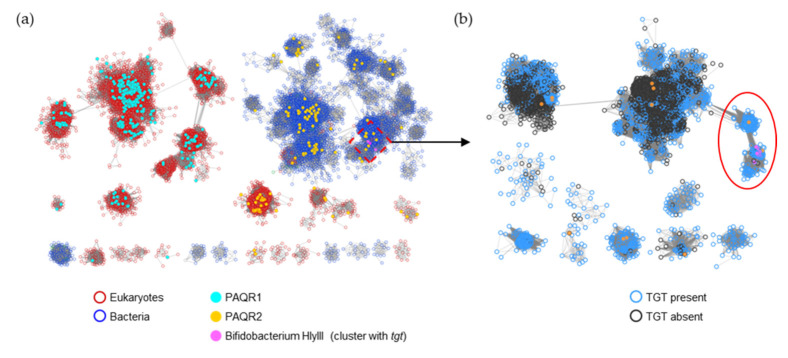
SSN of the HlyIII family (IPR004254/PF03006). (**a**) Each node in the network represents one or multiple HlyIII proteins that share no less than 50% identity. An edge (represented as a line) is drawn between two nodes with a BLAST E-value cutoff of better than 10^–45^ (alignment score threshold of 45). Node borders were colored by superkingdom. The PAQR groups as classified in [34] and *Bifidobacterium* HlyIII proteins encoded by genes that are next to *tgt* are colored. The HlyIII members connected to the preQ_1_ transporter in *B. breve* were further analyzed *(*boxed). (**b**) Each node in the network represents one HlyIII protein connected to the preQ_1_ transporter in *B. breve* (A0A0M3T8W5) as boxed in (**a**). An edge is drawn between two nodes with an alignment score better than 65. The nodes in the red circle are predicted to be the preQ_1_ transporters. For better visualization, the solitary nodes and small clusters are hidden. Node borders are colored by the presence (blue) or absence (black) of bTGT.

HlyIII (IPR004254/PF03006) is a family of ~48,000 proteins with a wide variety of annotated functions. Previous analysis of the family detailed several subgroups, including PAQR1 and PAQR2 [34]. PAQR1 only consists of eukaryotic proteins and includes the originally identified adiponectin receptor of mammals ([34] and Figure 9a). In contrast, PAQR2 consists of both bacterial and eukaryotic proteins (Figure 9a). The bacterial homologs are frequently annotated as ‘hemolysin III’, but, as it concerns any experimental validation, the functions of members of this PAQR2 subgroup remain unknown [34]. The *B. breve* HlyIII member found to transport preQ_0_/preQ_1_ is part of this larger PAQR2 subgroup (Figure 9a). Further analysis, here, of the proteins linked by similarity to the experimentally validated *B. breve* preQ_1_ transporter implies that only a subset (circled in Figure 9b and listed in Table S4) are likely to share this function, as most of the HlyIII proteins in surrounding subclusters originate from organisms that do not encode bTGT (black-circled nodes in Figure 9b).

**Figure 10:**
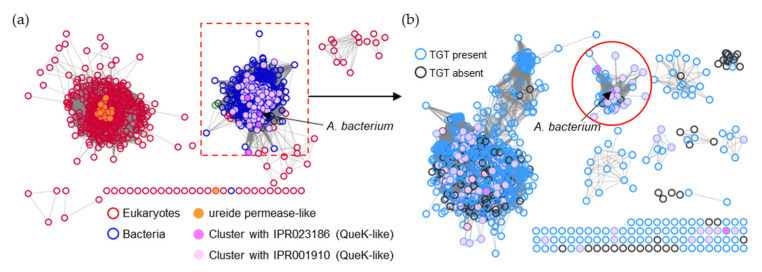
SSN of the ureide permease family (IPR009834/PF07168). (**a**) Each node in the network represents one ureide permease protein. An edge (represented as a line) is drawn between two nodes with a BLAST E-value cutoff of better than 10^–30^ (alignment score threshold of 30). Node boarders were colored by superkingdom. Members that share no less than 90% identity with curated ureide permeases in the Uniprot database are colored in orange. Nodes encoded by genes that cluster with *queK-*like genes are colored in pink. The UPS members connected to the preQ_1_ transporter in the *Acidobacteriota* bacterium (boxed) were further analyzed. (**b**) Each node in the network represents one UPS protein connected to the preQ_1_ transporter in the *Acidobacteriota* bacterium (A0A2V9U0M9) boxed in (**a**). An edge is drawn between two nodes with an alignment score better than 100. Node borders are colored by the presence (blue) or absence (black) of bTGT. The nodes in the red circle are predicted to be the preQ_1_ transporters.

The ureide permease (IPR009834/PF07168) identified here is a family of ~2000 proteins thought to transport a wide spectrum of oxo-derivative heterocyclic nitrogen compounds, including allantoin, uric acid, and xanthine [35,36,37]. The UPS member found to transport preQ_0_/preQ_1_ in the *Acidobacteriota* bacterium (A0A2V9U0M9) separates well from other UPS members that have been characterized in plants (Figure 10a). In this case, we can confidently propagate this annotation to the defined subset of UPS proteins indicated in Figure 10b and listed in Table S4. However, this annotation should be spread no further as, again, many UPS proteins in the other subclusters are from organisms that do not encode bTGT proteins (black-circled nodes in Figure 10b).

The Major Facilitator Superfamily (IPR011701/PF07690) (MFS) examined here is a family with more than 4 million members, representing the largest family of secondary transporters with members originating everywhere, from Archaea to *Homo sapiens* [38,39]. Many of the characterized MFS subgroups transporting a variety of compounds were found to separate well in the initial SSN (Figure S6). However, because of the family’s size, it was only possible to sample its entirety for our analyses (1/100 sequences at every node) and, as a result, lacked the necessary resolution to confidently predict members expected to be isofunctional with the *B. henselae* MFS transporter found to transport preQ_0_/preQ_1_, even if we had been able to extract the subgroup (circled in Figure S6 insert) of sequences only from genomes also encoding bTGT (blue-circled nodes in Figure S6 and Table S4).

## 3. Conclusions

Phylogenomic investigations into the Q synthesis and salvage genes in microbiome species spanning various biogeographical regions indicate that precursors of queuosine (preQ_0_, preQ_1_, q, and Q) could influence microbiome community composition at the species level [40]. Moreover, these precursors could potentially play a role in enhancing the host’s overall health. Therefore, queuosine and its precursors should be included in the roster of metabolites examined in forthcoming microbiome research.

It is well established that plasticity drives the evolution of novel transporters from existing ones [41,42,43,44,45], but our results also show that very different transporters can evolve to transport the same preQ_1_ molecule. Indeed, the five experimentally validated preQ_1_ transporters are members of four different transporter superfamilies according to the TCDB database [46] classification using the Transporter Class (TC) numbering system: the Vitamin Uptake Transporter (VUT) Family (TC# 2.A.88), the Major Facilitator Superfamily (MFS; TC# 2.A.1), the hemolysin III (Hly III) family (TC# 1.C.113), and the Drug/Metabolite Transporter (DMT) Superfamily (TC# 2.A.7). The bioinformatic analyses performed in the current study suggest that more transporters remain to be discovered and/or validated, including the other candidates that were identified and not yet tested (Table 1).

**Figure 11:**
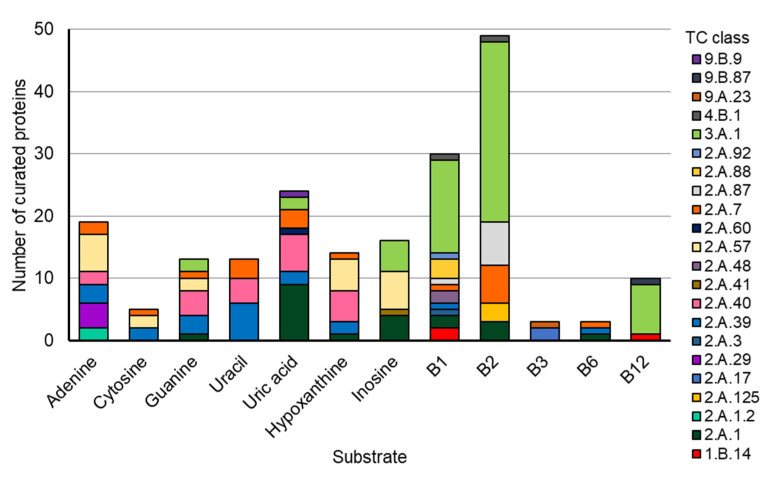
The composition of TCs for the common bases and B vitamins (summarization of Table S5). Data were retrieved for the indicated substrates using the Substrate Search Tool on the Transporter Classification Database (https://www.tcdb.org/ accessed on 26 February 2024).

This convergent evolution of members of different transporter families to transport the same molecule seems to be a recurrent evolutionary scenario, as it was previously seen for members of the bacterial Solute Binding Protein superfamily [47]. To further explore how common cases of convergent evolution among transporters may truly be, we used the new “molecule” search feature on the TCDB database [46], examining the number of different transporter families known to transport the common nucleobases and B vitamins (Figure 11 and Table S5). With this, it was observed that all molecules analyzed could be transported by members of at least 3 and up to 11 superfamilies.

Finally, this work reiterates the difficulty of predicting the substrate specificity of transporters based just on sequence similarity and the need to combine comparative genomics with experimental validation to functionally annotate this functional group. Systematic efforts to screen transporter substrate specificity are being implemented [48,49,50]. These will need to be combined with specialized transporter annotation capture and propagation tools to generally improve the current transporter databases [46,51].

## 4. Materials and Methods

### 4.1. Human Gut Microbiome Analysis

We retrieved 3632, 5387, and 4644 genome sequences of human gut bacterial and archaeal isolates and metagenome-assembled genomes from three comprehensive high-quality reference biobanks of the human gut microbiome, respectively: the Broad Institute-OpenBiome Microbiome Library (BIOML) [52], the Global Microbiome Conservancy (GMbC) [53], and the Unified Human Gastrointestinal Genome (UHGG) collection [54]. We retrieved 10 near-universal and single-copy ribosomal protein families (L2, L3, L4, L5, L6, L14, L16, L18, S3, and S8) using Diamond v0.8 (with BLASTx parameters: -more-sensitive -e 0.000001 -id 35 -query-cover 80) [55]. Each ribosomal protein family was aligned independently using MUSCLE v5.1 [56], trimmed using BMGE v1.12 (parameters: -t AA -g 0.95 -m BLOSUM30) [57], and concatenated using Seaview v4.751 [58]. The phylogenetic tree was reconstructed using FastTree v2.1 (parameters: -lg -gamma 20) [59] and was visualized and modified in iTOL v6.8 (https://itol.embl.de/ accessed on 30 January 2024) [60]. For better visualization, Orders with less than 10 genomes were hidden or merged. The presence of Q pathway genes was determined using tBLASTn [61] with thresholds of 20% and 1 × 10^−10^ for identity and E-value, respectively. The identifiers of query proteins of the Q pathway are listed in Table S6. The presence–absence pattern was visualized using an in-house program available at https://github.com/vdclab/published_scripts, accessed on 26 February 2024.

### 4.2. Human Oral Microbiome Analysis

The tree of human oral microbiome bacteria was adapted from the genomic tree version 10.1 of 8622 genomes in the expanded Human Oral Microbiome Database v3.1 (https://www.homd.org/ accessed on 19 February 2024). The tree was visualized and modified in iTOL (60). For better visualization, Orders with less than 10 genomes were hidden or merged. The presence of Q pathway genes was determined using BLASTp [61] with thresholds of 20% and 1 × 10^−10^ for identity and E-value, respectively.

### 4.3. Comparative Genomics and Sequence Similarity Networks (SSNs)

All bacterial fusion proteins of transporters and TGT were retrieved using the “similar architecture” tool of CDD (NCBI) [62], and with the “advanced search” tool of UniProt [63] (query: “Queuine tRNA-ribosyltransferase” AND (length:[500 TO 2000]) AND (taxonomy_id:2) AND transporter).

The classification of the different types of Q pathway profiles (e.g., de novo synthesis, q direct or indirect q salvage) in any given taxonomic id (Dataset S3), was based on the presence and absence of QueD, QueE, QueC, QueF, bTGT, QueA, and QueG/H proteins (as described in Figure 1). The presence of these proteins was inferred from the InterPro annotations in the corresponding genomes [64] using the InterPro family IDs listed in Table S6. These Q pathway profile characteristics were then used to color the node borders in the SSNs when stated.

As illustrated in Figure S1, SSNs were generated using the Enzyme Function Initiative (EFI) analytic suite [32] and visualized using Cytoscape (3.10.1) [65]. Sequences for each family, including IPR019438 (Qng1), IPR004803 (TGT), IPR023186 (QueK), IPR009834 (ureide permease), IPR011701 (MFS), and IPR004254 (AdipoR/Haemolysin-III-related), were retrieved using the “Family” option of EFI-EST (EFI Enzyme Similarity Tool). The initial SSN was generated with an alignment score (AS) cutoff set such that each connection (edge) represented a sequence identity above 40%. The specific node coloring patterns are given in the figure legends. More stringent SSNs were then created by gradually increasing the alignment score cutoff in small increments (usually by 5 AS units). This process was repeated until clusters were homogeneous in color. Edges were drawn between nodes with a BLAST E-value over the cutoff (alignment score threshold) as indicated in each SSN. The genomic neighborhoods were analyzed using EFI-GNT (Genome Neighborhood Tool) with a minimal co-occurrence filter set to 0 [32]. For neighborhood selection, the Pfam family of transporter candidates were selected with a median distance to bTGT, QueK, or Qng1 of no more than 2 and with a physical clustering ratio of more than 0.2% of total family members with recognized neighbors. The information on genomic regions was retrieved using EFI-GNT [32] and the gene neighborhood diagram was created using Gene Graphics [66]. The Transporter Classification Database [46] was used to further classify transporter families.

### 4.4. Strains, Media, and Growth Conditions

All strains and plasmids used in this study are listed in Table S7. LB medium (tryptone 10 g/L, yeast extract 5 g/L, sodium chloride 5 g/L) was routinely used for *E. coli* strain growth at 37 °C. The medium was solidified using 15 g/L of agar. As needed, kanamycin (50 µg/mL), ampicillin (100 µg/mL), and chloramphenicol (25 µg/mL) were added. In the presence of exogenous Q precursors as previously described [7,9], cells were cultured in M9-defined medium containing 1% glycerol (Thermo Fisher Scientific, Waltham, MA, USA). After cells reached an optical density at 600 nm (OD_600nm_) of 0.1–0.2, 0.2% arabinose was added to induce the expression of genes under the P_BAD_ promoter. After cells reached an OD_600nm_ of 0.2, DMSO, preQ_0_, preQ_1_, q, or Q were added. The transport reaction was stopped at time points of 30 or 60 min after supplementing with DMSO or different Q precursors by placing samples on melting ice and then centrifuging, followed by tRNA extraction. Q was purchased from Epitoire (Singapore), q from Santa Cruz Biotechnology (Dallas, TX, USA), and preQ_1_ and preQ_0_ from Sigma-Aldrich (St. Louis, MO, USA).

### 4.5. Construction of E. coli Strains and Plasmids

The genes encoding the candidate transporter proteins listed in Table 1 were chemically synthesized (without optimization) in pTWIST_Kan vectors (all DNA sequences are given in Table S8). The *Bifidobacterium breve* Hly_III, *Bacteroides henselae* MFS, *Corynebacterium propinquum* MmpL, *Winogradskyella*, and *Bacteroides dorei* YgjP encoding genes were directly subcloned from the corresponding pTWIST constructs into the EcoRI and HindIII sites of pBAD24 [67]. The *Chryseobacterium piperi* YgjP encoding gene was directly subcloned from the corresponding pTWIST construct into the EcoRI and PstI sites of pBAD24. The *Acidobacteriota* bacterium (Ac_UPS) gene was amplified from the pTWIST clones using the following primer pairs: F_Ac_DMT_NheI_PBAD24/R_Ac_DMT_XbaI_PBAD24; then, it was cloned into the NheI and XbaI sites of pBAD24. *E. coli* transformations were performed using the CaCl_2_ chemical transformation procedure [68]. Transformants were selected on LB agar supplemented with ampicillin. The clones were validated through Sanger sequencing and PCR analyses of the plasmids extracted using QIAGEN (Germantown, MD, USA) plasmid Mini kits with the appropriate primer pairs. All primers used in this study are listed in Table S9.

### 4.6. Q Detection Assay

Cells were harvested by centrifugation at 16,000× *g* for 2 min at 4 °C. Immediately after pelleting, the cells were resuspended in 1 mL of Trizol (Thermo Fisher Scientific, Waltham, MA, USA). According to the manufacturer’s instructions, small RNA was extracted with a PureLink miRNA Isolation kit (Thermo Fisher Scientific, Waltham, MA, USA). Briefly, 25 μL of RNase-free water was used to elute the purified RNAs. Quantification of prepared tRNA was performed using a Nanodrop 1000 spectrophotometer (Thermo Fisher Scientific, Waltham, MA, USA). We loaded 500 ng of tRNAs per well on a denaturing 8 M urea, 8% polyacrylamide gel containing 0.5% 3-(Acrylamido) phenylboronic acid (APB) (Sigma-Aldrich) after resuspending in a 2X RNA Loading Dye (NEB). Migration was performed in 1X TAE running buffer using a BioRad Mini-PROTEAN system and run in a stirred ice bath at 120 constant volts. tRNAs were transferred onto a Biodyne B precut nylon membrane (Thermo Fisher Scientific, Waltham, MA, USA) with a BioRad Trans-Blot SD semi-dry transfer cell apparatus at 10 V for 15 min. The membrane was UV-irradiated in a UV crosslinker (Fisher FB-UVXL-1000, Melville, NY, USA) at a preset UV energy dosage of 120 mJ/cm^2^. A North2South Chemiluminescent Hybridization and Detection Kit (Thermo) was used to detect tRNA^Asp^. As the DIG Easy Hyb (Roche) drastically reduces the background noise, it was used as the initial membrane-blocking buffer instead of the North2South kit’s membrane-blocking buffer. Hybridization was performed at 61 °C, using the specific biotinylated primer for tRNA^Asp^_GUC_ (5′ biotin-CCCTGCGTGACAGGCAGG 3′ for *E. coli*) added to a final concentration of 50 ng/mL. The blot was visualized by iBright™ Imaging Systems.

## Figures and Tables

**Table 1:** Top Q precursor transporter candidates.

Pfam	Description	Experimentally Tested Candidates (Species and Accession Number)
PF07690	Major Facilitator Superfamily (MFS_1)	*Bartonella henselae (*A0A0H3LX18) (Bh_MFS)
PF03176	MMPL family (MMPL)	*Corynebacterium propoinquum* (0A2W5NLC3)
PF03006	Haemolysin-III-related (Hly_III)	*Brevibacterium breve* (A0A0M3T8W5) (Bb_HlyII)
PF13515	Fusaric acid resistance protein-like superfamily (FUSC_2)	Not tested
PF00892	EamA-like transporter family (EanA)	Not tested
PF02355	Protein export membrane protein (SecD/SecF)	Not tested
PF03739	Lipopolysaccharide export system permease (LptF/LptG)	*Winogradskyella* sp. (A0A024FC69) *Chryseobacterium piperi* (A0A086BN18) *Bacteroides* (*Phocaeicola*) *dorei* (A0A076J562)
PF06541	Putative ABC-transporter type IV (ABC_trans_CmpB)	Not tested
PF07168	Ureide permease (UPS)	*Acidobacteriota* bacterium (A0A2V9U0M9) (Ac_UPS)
PF00005	ABC transporter (ABC_tran)	Not tested

## Data Availability

The original contributions presented in the study are included in the article/Supplementary Materials; further inquiries can be directed to the corresponding author/s.

## Supplementary Materials

The following supporting information can be downloaded at: https://www.mdpi.com/article/10.3390/epigenomes8020016/s1. Table S1: Candidate transporters from fusion searches in the UniProt database. Table S2: Candidate transporters from GNN analysis of TGT, Qng1, and QueK families. Table S3: Gene neighborhood information. Table S4: UniProt identifiers for predicted preQ_1_ transporter subgroups of the PF07168, PF03006, and PF07690 families. Table S5: Transporter families involved in the transport of bases and vitamins collected from https://www.tcdb.org/ accessed on 26 February 2024. Table S6: ID of query Q pathway proteins. Table S7: Strains and plasmids used in this study. Table S8: Information for synthesized genes. Table S9: Oligonucleotides used in this study. Dataset S1: Count of Q pathway genes in genomes of human gut microbiome collected from BIOML, GMbC, and UHGG. Dataset S2: Count of Q pathway genes in genomes of human oral microbiome that are collected in https://www.homd.org/ accessed on 19 February 2024. Dataset S3: Q pathway profile in given taxon based on Q synthesis and salvage proteins in UniProt database. Figure S1: The schematic representation of the comparative genomics analyses used in this study. Figure S2: Examples of physical clustering of transporter genes with Q pathway genes. Figure S3: YgjP of *Winogradskyella* sp. (Wi), *Bacteroides dorei* (Bd), and *Chryseobacterium piperi* (Cpi) and MMPL of *Corynebacterium propinquum* (Cpr) do not transport preQ_1_. Figure S4: *Acidobacteriota* bacterium (Ac) UPS, *Bifidobacterium breve* (Bb) HlyIII, and *Bartonella henselae* (Bh) MFS salvage preQ_1_ at low concentrations. Figure S5: UPS of *Acidobacteriot*a bacterium (Ac) cannot transport Q even at high concentrations but can transport q only at very high concentrations. Figure S6: SSN of the MFS family (IPR011701/PF07690).

## References

[1] Dedon P.C., Begley T.J. (2014). A system of RNA modifications and biased codon use controls cellular stress response at the level of translation. Chem. Res. Toxicol..

[2] Helm M., Motorin Y. (2017). Detecting RNA modifications in the epitranscriptome: Predict and validate. Nat. Rev. Genet..

[3] Huber S.M., Leonardi A., Dedon P.C., Begley T.J. (2019). The versatile roles of the tRNA epitranscriptome during cellular responses to toxic exposures and environmental stress. Toxics.

[4] Bohnsack M.T., Sloan K.E. (2017). The mitochondrial epitranscriptome: The roles of RNA modifications in mitochondrial translation and human disease. Cell Mol. Life Sci..

[5] Chan C., Pham P., Dedon P.C., Begley T.J. (2018). Lifestyle modifications: Coordinating the tRNA epitranscriptome with codon bias to adapt translation during stress responses. Genome Biol..

[6] de Crécy-Lagard V., Hutinet G., Cediel-Becerra J.D., Yuan Y., Zallot R., Chevrette M.G., Ratnayake R.M.N., Jaroch M., Quaiyum S., Bruner S. (2024). Biosynthesis and function of 7-deazaguanine derivatives in bacteria and phages. Microbiol. Mol. Biol. Rev..

[7] Yuan Y., Zallot R., Grove T.L., Payan D.J., Martin-Verstraete I., Šepić S., Balamkundu S., Neelakandan R., Gadi V.K., Liu C.F. (2019). Discovery of novel bacterial queuine salvage enzymes and pathways in human pathogens. Proc. Natl. Acad. Sci. USA.

[8] Hung S.H., Elliott G.I., Ramkumar T.R., Burtnyak L., McGrenaghan C.J., Alkuzweny S., Quaiyum S., Iwata-Reuyl D., Pan X., Green B.D. (2023). Structural basis of Qng1-mediated salvage of the micronutrient queuine from queuosine-5′-monophosphate as the biological substrate. Nucleic Acids Res..

[9] Zallot R., Yuan Y., de Crécy-Lagard V. (2017). The *Escherichia coli* COG1738 member YhhQ is involved in 7-cyanodeazaguanine (preQ_0_) Transport. Biomolecules.

[10] Ames B.N. (2018). Prolonging healthy aging: Longevity vitamins and proteins. Proc. Natl. Acad. Sci. USA.

[11] Varghese S., Cotter M., Chevot F., Fergus C., Cunningham C., Mills K.H., Connon S.J., Southern J.M., Kelly V.P. (2017). In vivo modification of tRNA with an artificial nucleobase leads to full disease remission in an animal model of multiple sclerosis. Nucleic Acids Res..

[12] Cirzi C., Dyckow J., Legrand C., Schott J., Guo W., Perez Hernandez D., Hisaoka M., Parlato R., Pitzer C., van der Hoeven F. (2023). Queuosine-tRNA promotes sex-dependent learning and memory formation by maintaining codon-biased translation elongation speed. EMBO J..

[13] Skolnick S.D., Greig N.H. (2019). Microbes and monoamines: Potential neuropsychiatric consequences of dysbiosis. Trends Neurosci..

[14] Rashad S., Al-Mesitef S., Mousa A., Zhou Y., Ando D., Sun G., Fukuuchi T., Iwasaki Y., Xiang J., Byrne S.R. (2024). Translational response to mitochondrial stresses is orchestrated by tRNA modifications. bioRxiv.

[15] Díaz-Rullo J., González-Pastor J.E. (2023). tRNA queuosine modification is involved in biofilm formation and virulence in bacteria. Nucleic Acids Res..

[16] Magnúsdóttir S., Ravcheev D., de Crécy-Lagard V., Thiele I. (2015). Systematic genome assessment of B-vitamin biosynthesis suggests cooperation among gut microbes. Front. Genet..

[17] Rodionov D.A., Arzamasov A.A., Khoroshkin M.S., Iablokov S.N., Leyn S.A., Peterson S.N., Novichkov P.S., Osterman A.L. (2019). Micronutrient re-quirements and sharing capabilities of the human gut microbiome. Front. Microbiol..

[18] Yan F., Xiang S., Shi L., Zhu X. (2024). Synthesis of queuine by colonic gut microbiome via cross-feeding. Food Front..

[19] Kesh K., Mendez R., Mateo-Victoriano B., Garrido V.T., Durden B., Gupta V.K., Oliveras Reyes A., Merchant N., Datta J., Banerjee S. (2022). Obesity enriches for tumor protective microbial metabolites and treatment refractory cells to confer therapy resistance in PDAC. Gut Microbes.

[20] Varriale L., Coretti L., Dipineto L., Green B.D., Pace A., Lembo F., Menna L.F., Fioretti A., Borrelli L. (2022). An outdoor access period improves chicken cecal microbiota and potentially increases micronutrient biosynthesis. Front. Vet. Sci..

[21] Mark Welch J.L., Rossetti B.J., Rieken C.W., Dewhirst F.E., Borisy G.G. (2016). Biogeography of a human oral microbiome at the micron scale. Proc. Natl. Acad. Sci. USA.

[22] McCallum G., Tropini C. (2024). The gut microbiota and its biogeography. Nat. Rev. Microbiol..

[23] Mondragón-Palomino O., Poceviciute R., Lignell A., Griffiths J.A., Takko H., Ismagilov R.F. (2022). Three-dimensional imaging for the quantification of spatial patterns in microbiota of the intestinal mucosa. Proc. Natl. Acad. Sci. USA.

[24] Derrien M., Van Baarlen P., Hooiveld G., Norin E., Müller M., de Vos W.M. (2011). Modulation of mucosal immune re-sponse, tolerance, and proliferation in mice colonized by the mucin-degrader Akkermansia muciniphila. Front. Microbiol..

[25] Yasuda K., Oh K., Ren B., Tickle T.L., Franzosa E.A., Wachtman L.M., Miller A.D., Westmoreland S.V., Mansfield K.G., Vallender E.J. (2015). Biogeography of the intestinal mucosal and lumenal microbiome in the Rhesus macaque. Cell Host Microbe.

[26] Bowen W.H., Burne R.A., Wu H., Koo H. (2018). Oral biofilms: Pathogens, matrix, and polymicrobial interactions in microenvironments. Trends Microbiol..

[27] Sangha J.S., Barrett P., Curtis T.P., Métris A., Jakubovics N.S., Ofiteru I.D. (2024). Effects of glucose and lactate on *Streptococcus mutans* abundance in a novel multispecies oral biofilm model. Microbiol. Spectr..

[28] Pereira F.C., Berry D. (2017). Microbial nutrient niches in the gut. Environ. Microbiol..

[29] Gorkiewicz G., Moschen A. (2018). Gut microbiome: A new player in gastrointestinal disease. Virchows Archiv..

[30] Clarke G., Sandhu K.V., Griffin B.T., Dinan T.G., Cryan J.F., Hyland N.P. (2019). Gut reactions: Breaking down xenobiotic–microbiome interactions. Pharmacol. Rev..

[31] Henry C.S., Lerma-Ortiz C., Gerdes S.Y., Mullen J.D., Colasanti R., Zhukov A., Frelin O., Thiaville J.J., Zallot R., Niehaus T.D. (2016). Systematic identification and analysis of frequent gene fusion events in metabolic pathways. BMC Genom..

[32] Zallot R., Oberg N., Gerlt J.A. (2019). The EFI web resource for genomic enzymology tools: Leveraging protein, genome, and metagenome databases to discover novel enzymes and metabolic pathways. Biochemistry.

[33] Quaiyum S., Yuan Y., Sun G., Ratnayake R.M.M.N., Hutinet G., Dedon P.C., Minnick M.F., de Crécy-Lagard V. (2023). Queuosine salvage in *Bartonella henselae* Houston 1: A unique evolutionary path. bioRxiv.

[34] Pei J., Millay D.P., Olson E.N., Grishin N.V. (2011). CREST—A large and diverse superfamily of putative transmembrane hydrolases. Biol. Direct..

[35] Desimone M., Catoni E., Ludewig U., Hilpert M., Schneider A., Kunze R., Tegeder M., Frommer W.B., Schumacher K. (2002). A novel superfamily of transporters for allantoin and other oxo derivatives of nitrogen heterocyclic compounds in *Arabidopsis*. Plant Cell..

[36] Schmidt A., Baumann N., Schwarzkopf A., Frommer W.B., Desimone M. (2006). Comparative studies on ureide permeases in Arabidopsis thaliana and analysis of two alternative splice variants of AtUPS5. Planta.

[37] Schmidt A., Su Y.H., Kunze R., Warner S., Hewitt M., Slocum R.D., Ludewig U., Frommer W.B., Desimone M. (2004). UPS1 and UPS2 from *Arabidopsis* mediate high affinity transport of uracil and 5-fluorouracil. J. Biol. Chem..

[38] Sauve S., Williamson J., Polasa A., Moradi M. (2023). Ins and outs of rocker switch mechanism in major facilitator superfamily of transporters. Membranes.

[39] Quistgaard E.M., Löw C., Guettou F., Nordlund P. (2016). Understanding transport by the major facilitator superfamily (MFS): Structures pave the way. Nat. Rev. Mol. Cell Biol..

[40] Donia M.S., Fischbach M.A. (2015). Small molecules from the human microbiota. Science.

[41] Krypotou E., Evangelidis T., Bobonis J., Pittis A.A., Gabaldón T., Scazzocchio C., Mikros E., Diallinas G. (2015). Origin, diversification and substrate specificity in the family of NCS1/FUR transporters. Mol. Microbiol..

[42] Jørgensen M.E., Xu D., Crocoll C., Ernst H.A., Ramírez D., Motawia M.S., Olsen C.E., Mirza O., Nour-Eldin H.H., Halkier B.A. (2017). Origin and evolution of transporter substrate specificity within the NPF family. eLife.

[43] Kourkoulou A., Pittis A.A., Diallinas G. (2018). Evolution of substrate specificity in the Nucleobase-Ascorbate Transporter (NAT) protein family. Microb. Cell..

[44] Gournas C., Athanasopoulos A., Sophianopoulou V. (2018). On the evolution of specificity in members of the yeast amino acid transporter family as parts of specific metabolic pathways. Int. J. Mol. Sci..

[45] Teichmann L., Chen C., Hoffmann T., Smits S.H.J., Schmitt L., Bremer E. (2017). From substrate specificity to promiscuity: Hybrid ABC transporters for osmoprotectants. Mol. Microbiol..

[46] Saier M.H., Reddy V.S., Moreno-Hagelsieb G., Hendargo K.J., Zhang Y., Iddamsetty V., Lam K.J.K., Tian N., Russum S., Wang J. (2021). The Transporter Classification Database (TCDB): 2021 Update. Nucleic Acids Res..

[47] Cerna-Vargas J.P., Sánchez-Romera B., Matilla M.A., Ortega Á., Krell T. (2023). Sensing preferences for prokaryotic solute binding protein families. Microb. Biotechnol..

[48] Stanchev L.D., Møller-Hansen I., Lojko P., Rocha C., Borodina I. (2023). Screening of *Saccharomyces cerevisiae* metabolite transporters by 13C isotope substrate labeling. Front. Microbiol..

[49] Pochini L., Galluccio M. (2022). Heterologous (Over) Expression of human SoLute Carrier (SLC) in yeast: A well-recognized tool for human transporter function/structure studies. Life.

[50] Fernández M., Rico-Jiménez M., Ortega Á., Daddaoua A., García García A.I., Martín-Mora D., Mesa Torres N., Tajuelo A., Matilla M.A., Krell T. (2019). Determination of ligand pro-files for *Pseudomonas aeruginosa* Solute Binding Proteins. Int. J. Mol. Sci..

[51] Elbourne L.D.H., Tetu S.G., Hassan K.A., Paulsen I.T. (2017). TransportDB 2.0: A database for exploring membrane transporters in sequenced genomes from all domains of life. Nucleic Acids Res..

[52] Poyet M., Groussin M., Gibbons S.M., Avila-Pacheco J., Jiang X., Kearney S.M., Perrotta A.R., Berdy B., Zhao S., Lieberman T.D. (2019). A library of human gut bacterial isolates paired with longitudinal multiomics data enables mechanistic microbiome research. Nat. Med..

[53] Groussin M., Poyet M., Sistiaga A., Kearney S.M., Moniz K., Noel M., Hooker J., Gibbons S.M., Segurel L., Froment A. (2021). Elevated rates of horizontal gene transfer in the industrialized human microbiome. Cell.

[54] Almeida A., Nayfach S., Boland M., Strozzi F., Beracochea M., Shi Z.J., Pollard K.S., Sakharova E., Parks D.H., Hugenholtz P. (2021). A unified catalog of 204,938 reference genomes from the human gut microbiome. Nat. Biotechnol..

[55] Buchfink B., Xie C., Huson D.H. (2015). Fast and sensitive protein alignment using DIAMOND. Nat. Methods.

[56] Edgar R.C. (2004). MUSCLE: Multiple sequence alignment with high accuracy and high throughput. Nucleic Acids Res..

[57] Criscuolo A., Gribaldo S. (2010). BMGE (Block Mapping and Gathering with Entropy): A new software for selection of phylogenetic informative regions from multiple sequence alignments. BMC Evol. Biol..

[58] Gouy M., Guindon S., Gascuel O. (2010). SeaView Version 4: A multiplatform graphical user interface for sequence alignment and phylogenetic tree building. Mol. Biol. Evol..

[59] Price M.N., Dehal P.S., Arkin A.P. (2010). FastTree 2—Approximately maximum-likelihood trees for large alignments. PLoS ONE.

[60] Letunic I., Bork P. (2021). Interactive tree of life (iTOL) v5: An online tool for phylogenetic tree display and annotation. Nucleic Acids Res..

[61] Altschul S.F., Madden T.L., Schaffer A.A., Zhang J., Zhang Z., Miller W., Lipman D.J. (1997). Gapped BLAST and PSI-BLAST: A new generation of protein database search programs. Nucleic Acids Res..

[62] Marchler-Bauer A., Derbyshire M.K., Gonzales N.R., Lu S., Chitsaz F., Geer L.Y., Geer R.C., He J., Gwadz M., Hurwitz D.I. (2015). CDD: NCBI’s conserved domain database. Nucleic Acids Res..

[63] UniProt Consortium (2023). UniProt: The Universal Protein Knowledgebase in 2023. Nucleic Acids Res..

[64] Paysan-Lafosse T., Blum M., Chuguransky S., Grego T., Pinto B.L., Salazar G.A., Bileschi M.L., Bork P., Bridge A., Colwell L. (2023). InterPro in 2022. Nucleic Acids Res..

[65] Shannon P., Markiel A., Ozier O., Baliga N.S., Wang J.T., Ramage D., Amin N., Schwikowski B., Ideker T. (2003). Cytoscape: A software environment for integrated models of biomolecular interaction networks. Genome Res..

[66] Harrison K.J., de Crécy-Lagard V., Zallot R. (2018). Gene Graphics: A genomic neighborhood data visualization web application. Bioinformatics.

[67] Guzman L.M., Belin D., Carson M.J., Beckwith J. (1995). Tight regulation, modulation, and high-level expression by vectors containing the arabinose PBAD promoter. J. Bacteriol..

[68] Green R., Rogers E.J. (2013). Transformation of chemically competent *E. coli*. Methods in Enzymology.

