# Supplementary material for: Deciphering the Diversity in Bacterial Transporters That Salvage Queuosine Precursors
Source: Epigenomes. 2024 Apr 25;8(2):16. doi: 10.3390/epigenomes8020016 (PMC11130926; doi:10.3390/epigenomes8020016)
Supplement: Supplementary file 1 [file epigenomes-08-00016-s001.zip › Supplement figures_0304YY.pdf]

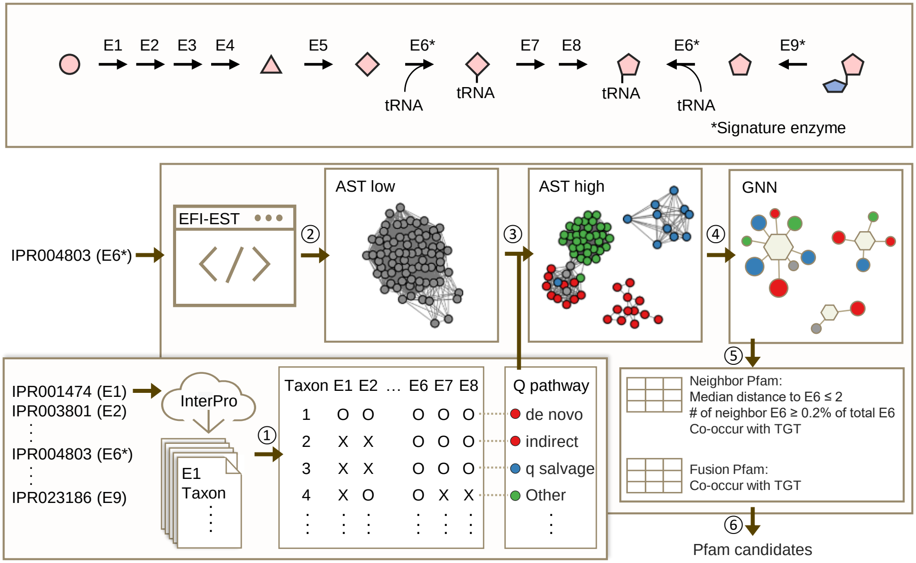

Figure S1. The schematic representation of comparative genomics analyses. (1) Preparation of the profile of Q pathway genes for each taxonomy IDs in InterPro database; (2) Construction of SSNs with a query enzyme, e.g. E6; (3) Coloring of SSNs with the profile of Q pathway and determination of AST that separates clusters by Q pathway types; (4) Generation of a Genome Neighborhood Network; (5) Selection of neighboring and fusion Pfams with criteria; (6) Selection of candidates. Enzymes in Q biosynthesis pathway are represented by E1 to E9. Among them, E6 (TGT) and E9 (hydrolases) are the signature enzymes of q salvage.

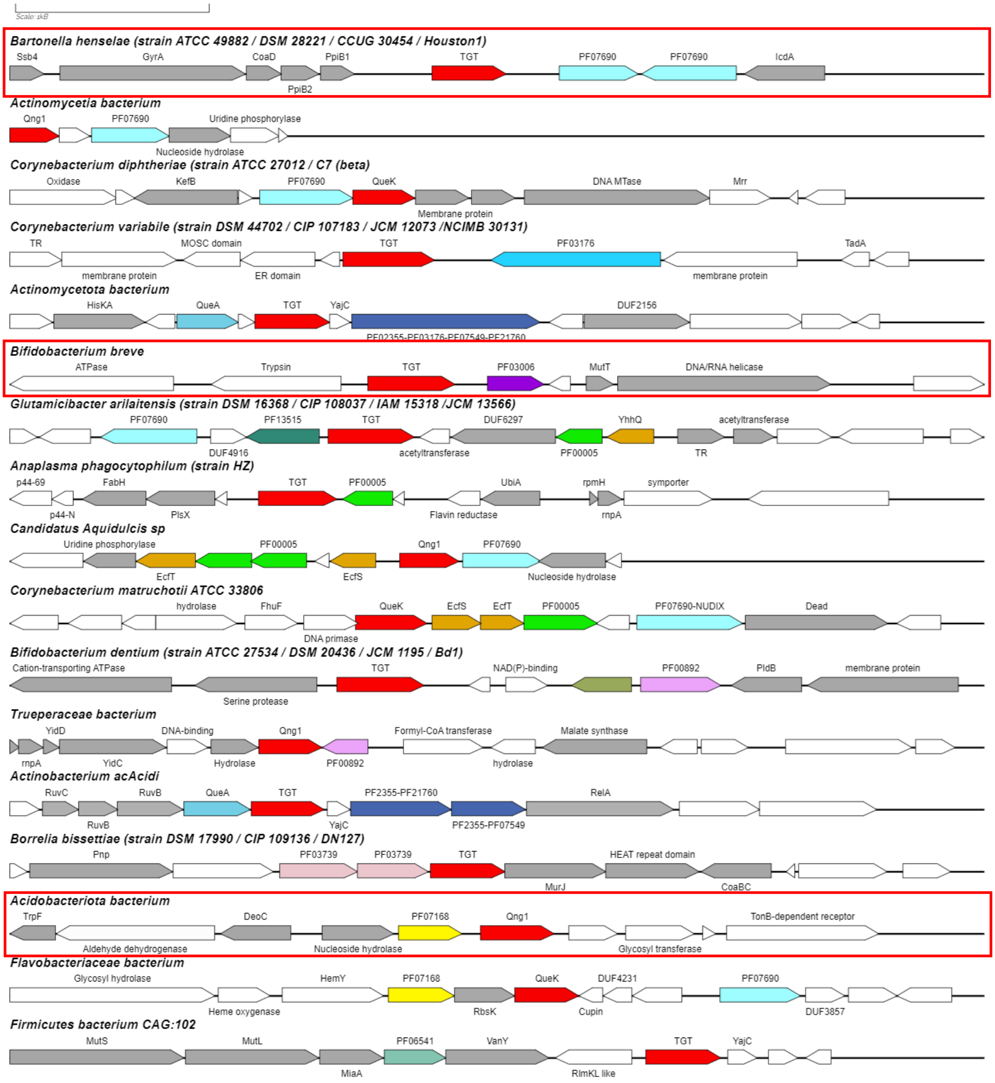

Figure S2. Examples of Physical clustering of transporter genes with the Q pathway genes. Organisms shown are indicated by arrows in SSNs. The information of genome regions and protein IDs are listed in Table S3. Organisms of which transporters have been tested in this study are boxed.

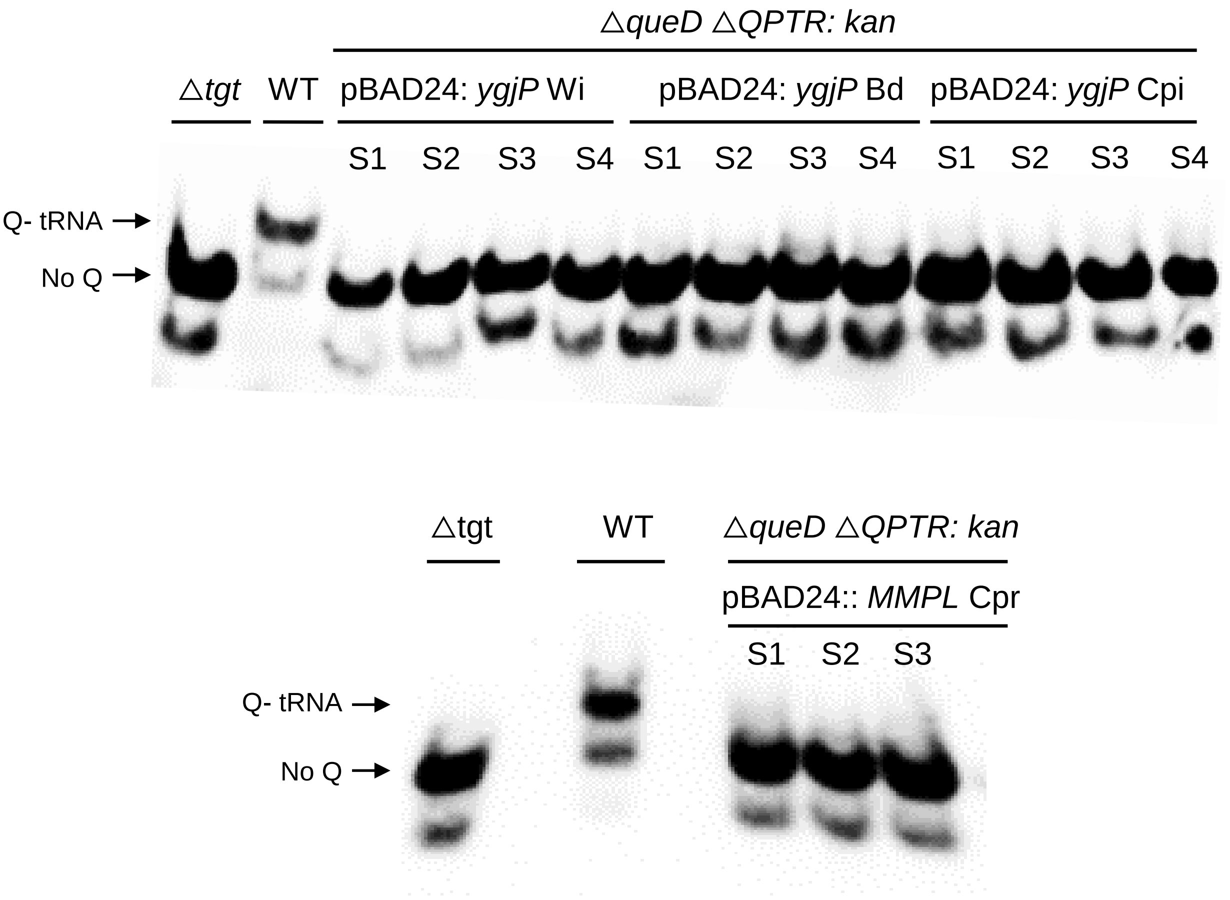

Figure S3. Testing the activities of YgjP of *Winogradskyella* sp. (Wi), *Bacteroides dorei* (Bd), *Chryseobacterium piperi* (Cpi), and MMPL of *Corynebacterium propinquum* (Cpr). Detection of $Q\text{-}tRNA^{Asp}_{GUC}$ by the APB assay. Q-modified tRNAs that migrated slower are indicated by an arrow. tRNAs were extracted from WT and different mutant strains expressing different Q salvage genes. The strains used are denoted in the first line. The genes and corresponding vectors are indicated in the second line. Plasmid and strain information is given in Table S7 and S8. Cells were grown a minimal media in the presence of 100nM exogenous $preQ_1$. S1, S2 and S3 represent biological replicates.

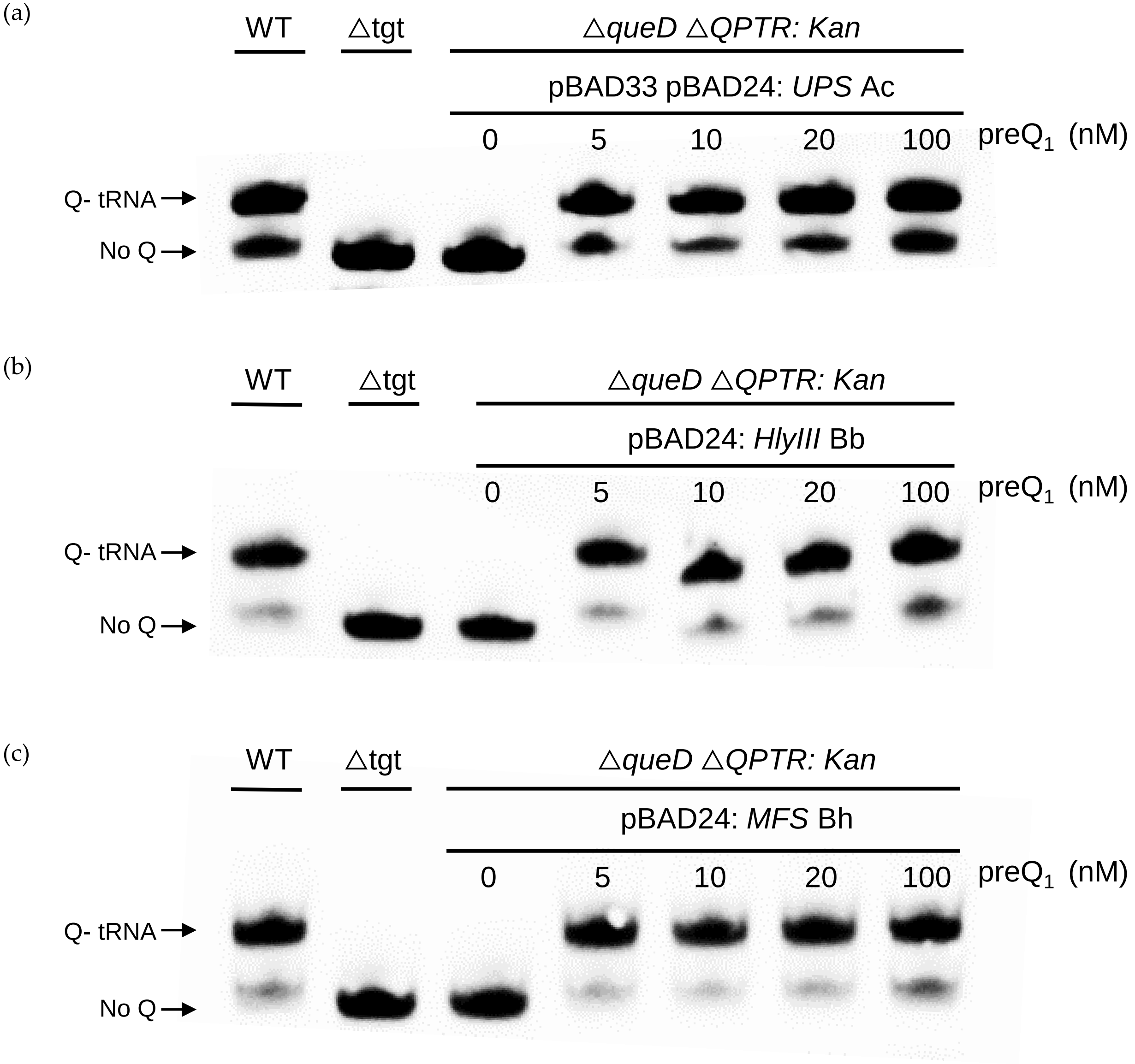

Figure S4. *Acidobacteriota bacterium* (Ac) UPS, *Bifidobacterium breve* (Bb) HlyIII and *Bartonella henselae* (Bh) MFS salvage preQ$_1$ at low concentrations. Detection of Q-tRNA^{Asp} $_{GUC}$ by the APB assay. Q-modified tRNAs that migrated slower are indicated by an arrow. tRNAs were extracted from WT and different mutant strains expressing different Q salvage genes that grew in minimal medium supplemented with various concentrations of preQ$_1$ (from 5 to 100 nM). The strains used are denoted in the first line. The genes and corresponding vectors are indicated in the second line. Plasmid and strain information is given in Table S7 and S8.

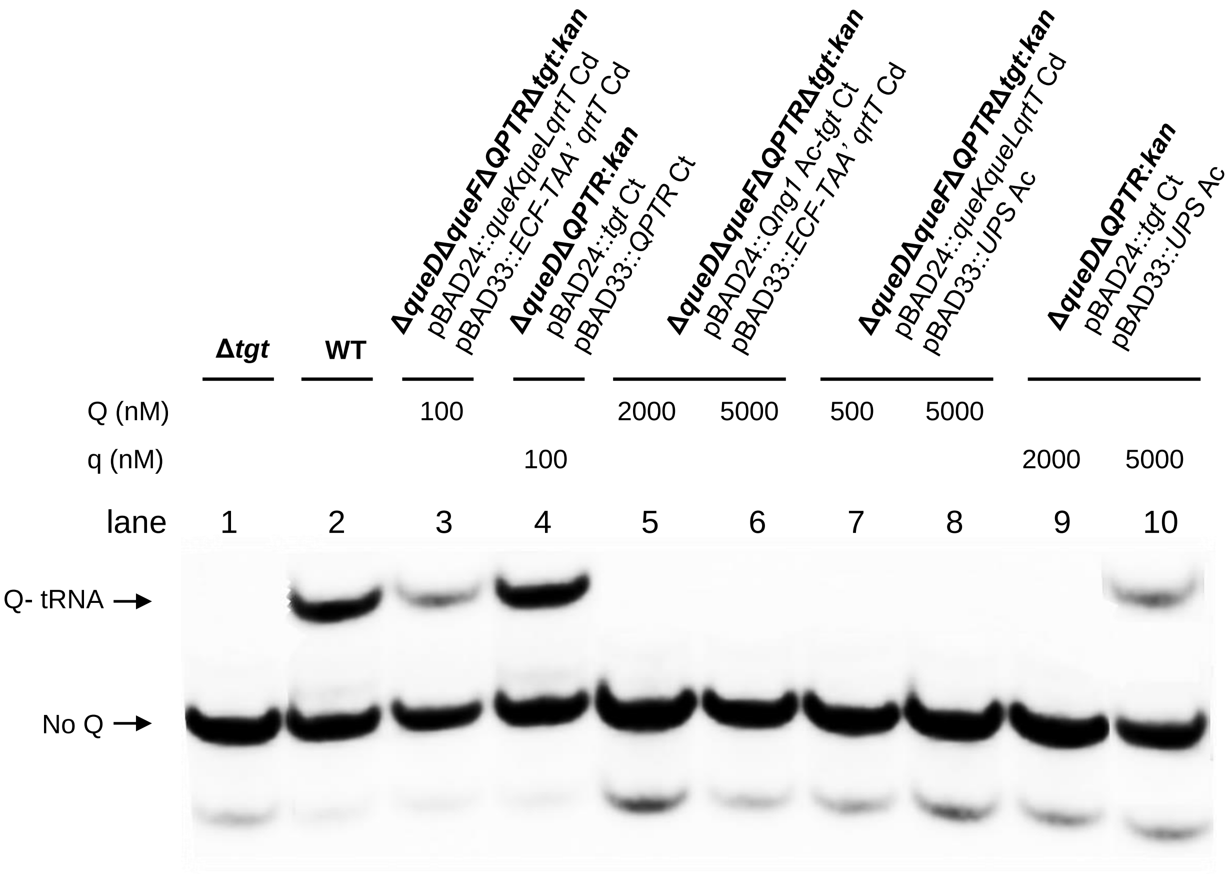

Figure S5. UPS of *Acidobacteria bacterium* (Ac) cannot transport Q even at high concentration like the ECF-QrtT of *Clostridioides difficile* (Cd) but can transport q only at very high concentrations. Detection of Q-tRNA$^{Asp}{}_{GUC}$ by the APB assay. Q-modified tRNAs that migrated slower are indicated by an arrow. tRNAs were extracted from WT and different mutant strains expressing different Q salvage genes that grew in minimal medium supplemented with various concentrations of q or Q (from 100 to 5000 nM). The strains used are in bold. Corresponding vectors and genes are denoted below strain names. Plasmid and strain information is given in Table S7 and S8. Lane 1 and 2 were used as negative and positive control for no Q- and Q- tRNA, respectively. Lane 3 and 4 were used as positive control for Q and q uptake and insertion, respectively. Qng1 Ac could not salvage Q like QueKL Cd (Lane 5 and 6). UPS Ac could transport q at high concentration (lane 9 and 10) but not Q (lane 7 and 8). *Chlamydia trachomatis* (Ct) QPTR and TGT specifically transports and inserts q in tRNA, respectively.

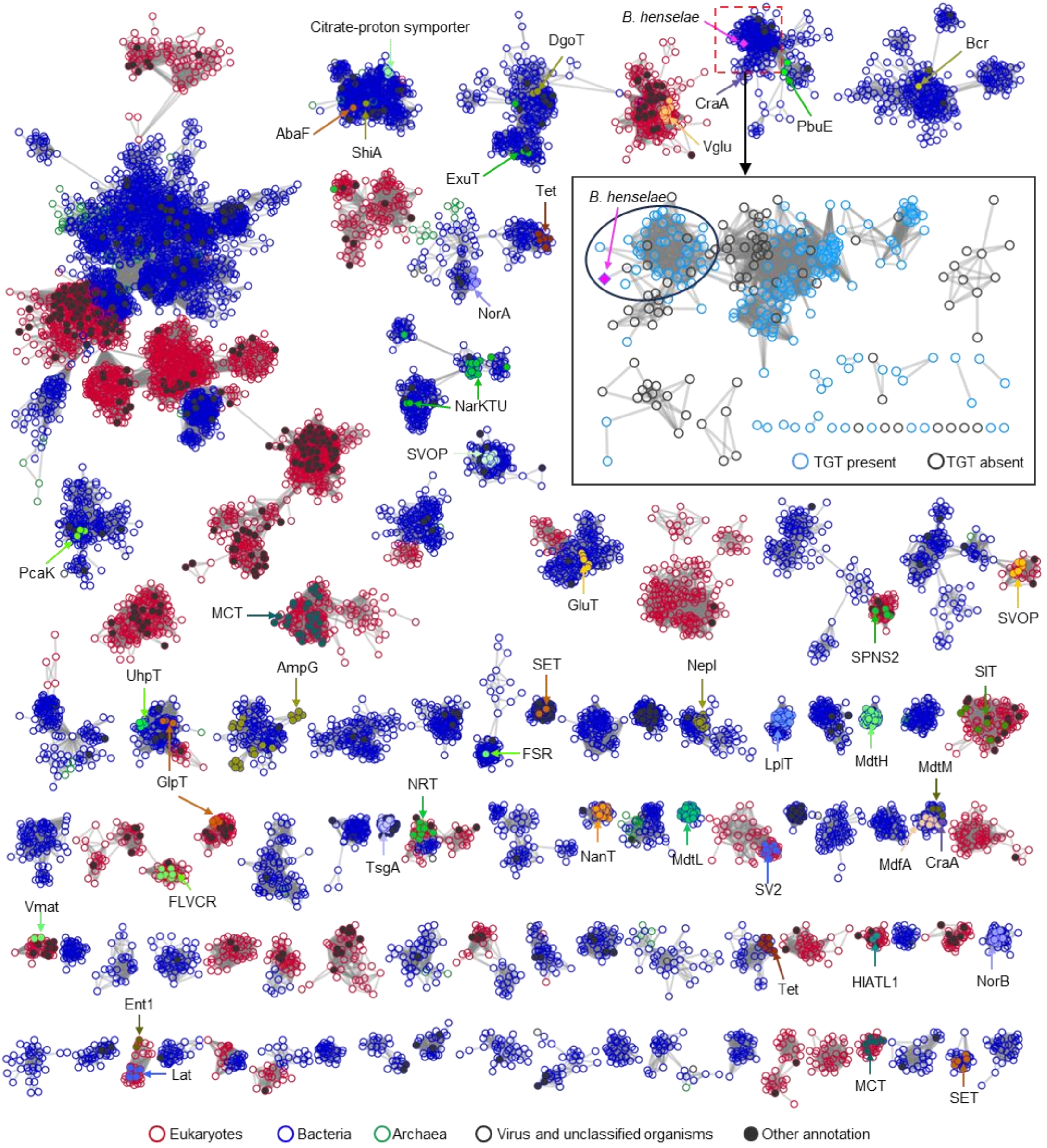

Figure S6. SSN of the MFS family (IPR011701 /PF07690). (A) Each node in the network represents one MFS protein that was selected from every 100th sequence in the family. An edge (represented as a line) is drawn between two nodes with a BLAST E-value cutoff of better than 10^{-50} (alignment score threshold of 50). Node boards were colored by superkingdom. Selected characterized members in Uniprot database were indicated. The MFS members connected to Q precursor transporter in *B. henselae* Houston 1 (A0A0H3LX18) (boxed) were further analyzed. (B) Each node in the network represents one MFS protein connected to Q precursor transporter in *B. henselae* Houston 1 (A0A0H3LX18) in previous SSN. An edge is drawn between two nodes with alignment score better than 79. Node boards were colored by the presence (blue) or absence (black) of TGT. For better visualization, the solitary nodes and small clusters are hidden.
